# Risk of work-related hand eczema in relation to wet work exposure

**DOI:** 10.5271/sjweh.3876

**Published:** 2020-07-01

**Authors:** Tamara Lund, Sesilje Bondo Petersen, Esben Meulengrath Flachs, Niels Erik Ebbehøj, Jens Peter Bonde, Tove Agner

**Affiliations:** Department of Dermatology, Bispebjerg University Hospital, Copenhagen, Denmark; Department of Occupational and Environmental Medicine, Bispebjerg University Hospital, Copenhagen, Denmark

**Keywords:** Key terms contact dermatitis, dermatitis, DOC*X, JEM, job exposure matrix

## Abstract

**Objective:**

Albeit a pivotal risk for the development of hand eczema (HE), the exposure–response relationship between wet work and HE remains to be further investigated. Knowledge on exposure–response is important regarding preventive measures, medico-legal regulations and job-counseling. Recently, a job-exposure matrix (JEM) for wet work was developed, providing information on the likelihood of wet work. By combining the JEM with data on HE we aimed to investigate the relationship between extent of wet work and HE.

**Methods:**

This study is a case–referent study including patients registered in the National Database of Contact Allergy, Denmark, and comprises data on sex, age, atopic dermatitis, HE, face eczema and patch testing results. Patients with HE served as cases and patients with facial eczema served as referents. Information on profession was retrieved from the DOC*X database in accordance with the DISCO-88 classification system. A wet-work-specific JEM provides – for each profession – an estimate for (i) the likelihood of wet work lasting ≥2 hours/day and (ii) the average number of hours of wet work per day.

**Results:**

After two hours of wet hands and glove wear, the odds ratio (OR) was 3.49 and 3.19, respectively, for females and 2.41 and 1.82, respectively, for males. Females had a higher risk of HE than males with probability of wet hands <75% (OR 2.34, 95% CI 2.12–2.58 compared to males 1.68, 95% CI 1.22–2.31) and regarding glove wear at all exposure levels.

**Conclusion:**

Our data confirms a close association between wet work and HE. Exposure lasting less than the current definition of wet work (having wet hands for ≥2 hours per day) may be of importance.

Wet work is one of the strongest known risk exposures for the development of work-related hand eczema (HE) (1–3), which is ranked among the top notified occupational diseases in several European countries, revealing a large potential for successful prevention strategies (3–6). Although wet work is a pivotal risk factor for developing work-related HE, the exposure–response relationship between extent of wet work and development of work-related HE remains to be further investigated (7, 8). Prior studies have shown that decreasing intensity or ceasing wet work has a significantly positive effect on the severity of work-related HE (9, 10). The definition of wet work as having wet hands for ≥2 hours per working day, hand washing ≥20 times per working day, or wearing occlusive gloves for ≥2 hours per working day is widely accepted; however, it does not take into account variations related to occupations or sex (11). Assuming a specific level of exposure representing an entire specific profession may disregard considerable individual variations among job tasks and sex. The variation among exposure levels between females and males have been documented in several studies, where females are exposed to higher levels of wet work than their male colleagues (12–14).

Variations in duration and frequency of wet work activities has been studied in specific wet work occupations, such as hairdressers, cleaners and health care workers; however, there are few studies regarding dose–response relationship (7, 10, 15–22). Knowledge on exposure-response is important regarding specific preventive measures, and also in relation to medico-legal regulations and individual job-counseling. Recently a job-exposure matrix (JEM) for wet work has been developed, providing information on the likelihood of wet-work activity (23). By combining data from the JEM with data on HE, in this study we aim to investigate the relationship between extent of wet work and diagnoses of HE.

## Methods

### Study population

This study is a case–referent study including patients registered in the National Database of Contact Allergy, Denmark (24). The database was founded in October 2002 and comprises data from patients who have been patch tested at a varying number of dermatological hospital departments (N=3–5) and private dermatology practices in Denmark (N=7–13). Data in this study covers the period 1 January 2003 to 31 December 2015. Data registered in the database comprise sex, age, status of atopic dermatitis (current or previous), HE, face eczema as well as result of patch testing (positive or negative). Patients identified with HE served as cases, and patients identified with face eczema served as referents.

### Assessment of profession

Information on profession was retrieved from the DOC*X database at Statistics Denmark (25). The database covers all employed Danish citizens from the age of 15 years and comprises information regarding annual status of profession, educational level, income level, resident children ≤4 years of age and, residence (25). Data on profession is categorized in accordance with the Danish DISCO-88 classification system based on the four-digit International Standard Classification of Occupations (ISCO)-88 classification system. Data from the National Database of Contact Allergy, Denmark, was linked at the individual level with data from the database DOC*X at Statistics Denmark using the Danish personal identification number (20), and the registered profession from the year prior to being included in the database was used. Likelihood of smoking was estimated based on a sex, age and calendar year specific JEM addressing lifestyle factors, such as tobacco smoking (26).

### Exposure assessment

Exposure to wet-hand activities was assigned by a wet-work-specific JEM, based on a self-reported question about wet-hand activity from national surveys on working environment performed by the National Research Centre for the Working Environment in Denmark in 2000, 2005, and 2010 (National Research Centre for the Working Environment) (23). In the JEM, wet hands are defined as having wet or moist hands, and glove wear is defined as wearing protective gloves made of plastic or rubber. The JEM is based on 432 professions classified according to the DISCO-88 system and provides both an estimate for the likelihood of having wet hands or wearing gloves ≥2 hours/day for each profession and an estimate of the average number of hours per work day (8 hours) having wet hands/wearing gloves, respectively. Both variables are calculated for working hours only and do not include leisure-time activity. The estimates were calculated for each of the 432 professions by fitting a logistic model in SAS V.9.4 (SAS Institute, Cary NC, USA). For the purpose of this study, we linked estimates from the JEM to each individual in the study population by the DISCO-88 code from the DOC*X database.

### Outcome assessment

Outcome data included diagnosis of HE from the National Database of Contact Allergy, Denmark. Differentiation between different subgroups of HE is not considered. The database has patients registered in the MOAHLFA index (27) by dermatologists only, thus the diagnose of HE is assumed to be precise and reliable.

### Statistical analysis

**Figure 1:**
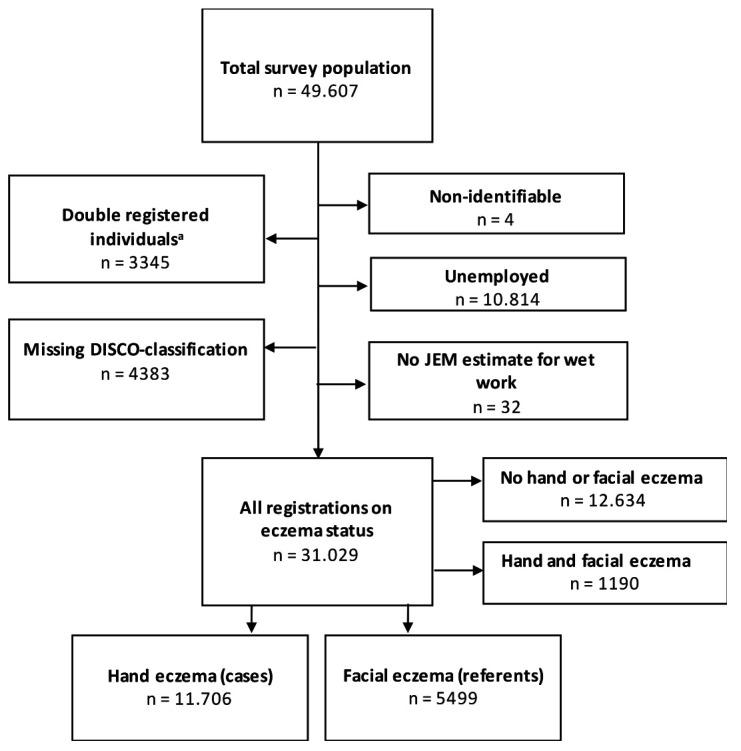
Flow chart of the selection of the study population from the National Database of Contact Allergy. * Individuals listed as double-registered covers individuals who have been patch-tested twice and therefore are represented more than once in the database.

Before performing any analysis, patients with combined HE and facial eczema were excluded (figure 1). Crude and adjusted risk for HE according to wet-work exposure were computed by logistic regression, that also provides 95% confidence intervals (CI). In the analysis, likelihood of wet-work exposure for ≥2 hours per work shift was divided in four groups (0–25, >25–50, >50–75, >75%). We did equal analyses with the exposure variable “glove wear”. We furthermore performed the analyses stratified by sex. In the main analyses, we adjusted for sex (1=male, 2=female), age (1=<30, 2=30–39, 3=40–49, and ≥50 years), educational level (1=primary school, 2=upper secondary education, 3=vocational upper secondary education, 4=medium-cycle higher education, and 5=long-cycle higher education), income level (1=low, 2=medium, 3=high, and 4=very high), resident children ≤4 years of age (0=no, 1=yes), residence (1=Copenhagen area, 2=Zealand, 3=Funen, 4=Jutland) atopic dermatitis (0=no, 1=yes) and result of patch test (0=no, 1=yes). We also adjusted for smoking by use of a smoking JEM with estimates of likelihood of being a smoker (%) for each DISCO-88 code. The group-based estimates were linked to each individual in the study population by the same DISCO-88 group as the group classifying the wet exposure likelihood. The smoking JEM is described elsewhere (26).

Tables 2a, 2b and 2c, present crude and three analyses adjusted, respectively, for (i) all potential confounders, (ii) only demographic (sex, age, resident children ≤4 years), and (iii) both demographic and socio-economic (education, income, residence and smoking).

A supplementary logistic regression analysis model with exposure as average hours per working day modelled as a natural spline with three knots was used when graphically illustrating the dose–response relationship between number of estimated average daily exposure hours from the JEM and risk of HE for the outcomes “wet hands” and “glove wear”, respectively. All analyses were performed in SAS version 9.4. A significance level of 0.05 was used throughout.

## Results

In our final study population (N=49 706), 11 706 had been diagnosed with solely HE and 5499 with solely facial eczema (figure 1). Information regarding wet hands was available for 17 205 individuals and regarding glove wear on 15 241, both attained by linkage with the JEM. Table 1 presents demographic characteristics of HE and facial eczema patients, respectively. The characteristics of patients with HE or facial eczema were rather similar, with sex distribution differing the most; 63.7% of HE patients were female compared to 81.0% of facial eczema patients. More HE patients had resident children ≤4 years, a lower proportion of atopic dermatitis, lower level of education and lower proportion of very high income level.

**Table 1:** Demographics of the study population, including cases (hand eczema) and referents (facial eczema) in the National Database of Contact Allergy.

	Total (N=17 205)	Patients with hand eczema N=11 706		Patients with facial eczema N=5499	
		
	N	%	Mean	%	Mean
Sex					
Female	11 908	63.7		81.0	
Age (years)			38.7		40.7
<30	4371	27.1		21.7	
30–39	4244	25.8		22.2	
40–49	4331	23.4		28.9	
≥50	4259	23.6		27.2	
Atopic dermatitis	3626	20.3		22.7	
Positive patch test	6955	40.1		41.1	
Smoking					
Likelihood of being smoker		24.0		21.0	
Resident children					
≤4 years of age	3092	19.8		14.0	
Missing ^a^	93	0.6		0.4	
Residence					
Copenhagen area	5742	31.9		36.6	
Zealand	2810	15.9		17.2	
Funen	1969	11.6		11.1	
Jutland	6590	40.0		34.7	
Missing ^a^	94	0.6		0.4	
Educational level ^b^					
Primary school	4192	26.4		19.9	
Upper secondary	1794	9.3		12.7	
Vocational upper secondary	6201	38.3		31.1	
Medium-cycle higher	3777	19.6		26.9	
Long-cycle higher	1005	4.7		8.2	
Missing ^a^	236	1.5		1.1	
Income level					
Low income level	2031	12.5		10.4	
Medium income level	3429	20.4		18.9	
High income level	7598	45.7		40.9	
Very high-income level	2612	12.6		20.8	
Missing ^a^	1535	8.9		9.0	

a Missing: no information listed in the DOC*X database.

b Educational level refers to the highest completed level of education.

The odds ratios (OR) for having HE based on having wet hands or wearing gloves – both measured as probability of ≥2 hours per working day – are presented in table 2. HE was significantly related to both wet-work activities (wet hands and glove wear), and the significant association increased concurrently with likelihood of wet work. With >75% probability of wet hands, the OR was 2.97 (95% CI 2.57–3.43) compared to OR 1.44 (CI 1.30–1.60) with >25–50% probability of wet hands. With >75% probability of glove wear, the OR was 2.50 (95% CI 2.20–2.85) compared to OR 1.72 (95% CI 1.53–1.93) with >25–50% probability of glove wear (table 2a). As shown in tables 2b and 2c, females had higher risk of HE compared to men when probability of wet hands was <75% (males OR 1.68, 95% CI 1.22–2.31 compared to females’ OR 2.34, 95% CI 2.12–2.58), but males had higher risk of HE compared to females when probability of wet hands was >75% (males OR 3.52, 95% CI 1.76–7.05 compared to females OR 2.95, 95% CI 2.54–3.43). This concerns both crude and adjusted analyses. Regarding glove wear, females had higher risk of HE compared to men at all levels of glove wear when compared to the reference group.

**Table 2a:** Odds ratio (OR) and 95% confidence interval (95% CI) for having hand eczema according to the probability of ≥2 hours of wet work activity in cases (hand eczema) and referents (facial eczema) in the National Database of Contact Allergy (N total=17 205). [ref=reference.]

	Hand eczema (N)	Facial eczema (N)	OR crude (95% CI)	OR adjusted (95% CI) ^a^	OR adjusted (95% CI) ^b^
Probability of having wet hands ≥2hours/day (N=17 205) ^c^					
0–25%	5585	3640	ref	ref	ref
>25–50%	2001	673	1.94 (1.76–2.13)	1.44 (1.30–1.60)	1.66 (1.50–1.83)
>50–75%	2809	897	2.04 (1.87–2.22)	2.27 (2.07–2.49)	2.62 (2.40–2.87)
>75%	1311	289	2.96 (2.59–3.38)	2.97 (2.57–3.43)	3.81 (3.32–4.37)
Probability of wearing gloves at work ≥2 hours/day (N=15 241) ^d^					
0–25%	5557	3705	ref	ref	ref
>25–50%	1802	503	2.39 (2.15–2.66)	1.72 (1.53–1.93)	2.10 (1.88–2.35)
>50–75%	1524	485	2.09 (1.88–2.34)	2.23 (1.99–2.50)	2.64 (2.35–2.95)
>75%	1281	384	2.22 (1.97–2.51)	2.50 (2.20–2.85)	2.75 (2.43–3.11)

a Adjusted for the variables sex, age, educational level, income level, resident children age of ≤4, residence, atopic dermatitis, positive patch test and the estimate of smoking.

b Adjusted for the variables sex, age, resident children age of ≤4.

c The exposure categories in the analyses refer to the probability of ≥2 hours/day of either wet hands or glove wear.

d Missing data on glove wear for N=1964.

**Table 2b:** Odds ratio (OR) and 95% confidence interval (95% CI) for having hand eczema according to extent of wet work activity in females in cases (hand eczema) and referents (facial eczema) in the National Database of Contact Allergy, (N total=17 205). [ref=reference.]

	Hand eczema (N)	Facial eczema (N)	OR crude (95% CI)	OR adjusted (95% CI) ^a^	OR adjusted (95% CI) ^b^
Probability of having wet hands ≥2 hours/day (N=17 205) ^c^					
0–25%	3093	2889	ref	ref	ref
>25–50%	776	440	1.65 (1.45–1.87)	1.55 (1.35–1.78)	1.69 (1.49–1.93)
>50–75%	2425	845	2.68 (2.44–2.94)	2.34 (2.12–2.58)	2.67 (2.43–2.94)
>75%	1160	80	3.87 (3.36–4.45)	2.95 (2.54–3.43)	3.81 (3.31–4.39)
Probability of wearing gloves at work ≥2 hours/day (N=15 241) ^d^					
0–25%	3752	3240	ref	ref	ref
>25–50%	1026	374	2.37 (2.09–2.69)	1.87 (1.63–2.14)	2.33 (2.05–2.63)
>50–75%	1421	464	2.64 (2.36–2.97)	2.34 (2.07–2.63)	2.77 (2.47–3.11)
>75%	1255	376	2.88 (2.54–3.26)	2.61 (2.28–2.98)	2.86 (2.52–3.24)

a Adjusted for the variables age, educational level, income level, resident children age of ≤4, residence, atopic dermatitis, positive patch test and the estimate of smoking.

b Adjusted for the variables age, resident children age of ≤4.

c The exposure categories in the analyses refer to the probability of ≥2 hours/day of either wet hands or glove wear.

d Missing data on glove wear for N=1964.

**Table 2c:** Odds ratio (OR) and 95% confidence interval (95% CI) for having hand eczema according to extent of wet work activity in males in cases (hand eczema) and referents (facial eczema) in the National Database of Contact Allergy, (N total=17 205). [ref=reference.]

	Hand eczema (N)	Facial eczema (N)	OR crude (95% CI)	OR adjusted (95% CI) ^a^	OR adjusted (95% CI) ^b^
Probability of having wet hands ≥2 hours/day (N=17 205) ^c^					
0–25%	2492	751	ref	ref	ref
>25–50%	1225	233	1.58 (1.35–1.86)	1.28 (1.07–1.53)	1.59 (1.35–1.87)
>50–75%	384	52	2.22 (1.65–3.01)	1.68 (1.22–2.31)	2.15 (1.59–2.91)
>75%	151	9	5.06 (2.57–9.95)	3.52 (1.76–7.05)	4.63 (2.34–9.16)
Probability of wearing gloves at work ≥2 hours/day (N=5241) ^d^					
0–25%	1805	465	ref	ref	ref
>25–50%	776	129	1.55 (1.25–1.92)	1.31 (1.04–1.64)	1.53 (1.23–1.89)
>50–75%	103	21	1.26 (0.78–2.04)	1.11 (0.68–1.82)	1.26 (0.78–2.04)
>75%	26	8	0.84 (0.38–1.86)	0.59 (0.26–1.35)	0.78 (0.36–1.78)

a Adjusted for the variables age, educational level, income level, resident children ≤age of 4, residence, atopic dermatitis, positive patch test and the estimate of smoking.

b Adjusted for the variables age, resident children age of ≤4.

c The exposure categories in the analyses refer to the probability of ≥2 hours/day of either wet hands or glove wear.

c Missing data on glove wear for N=1964.

**Figure 2:**
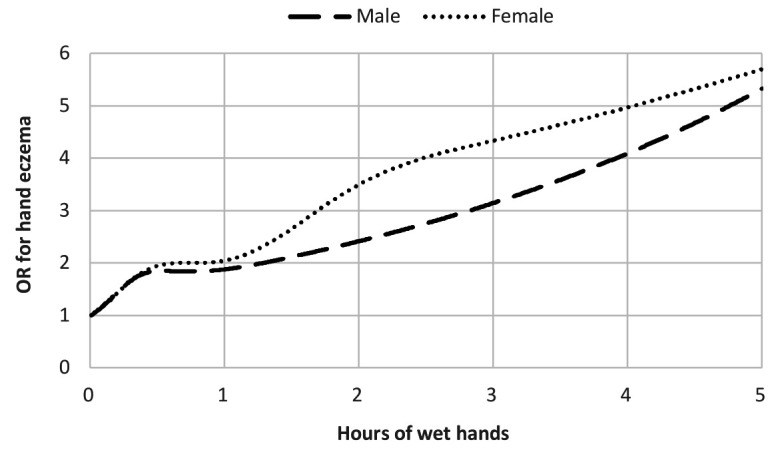
Risk (odds ratio) of hand eczema in men (N=5297) and women (N=11 908) according to a job exposure matrix derived estimate of average daily hours working with wet hands

**Figure 3:**
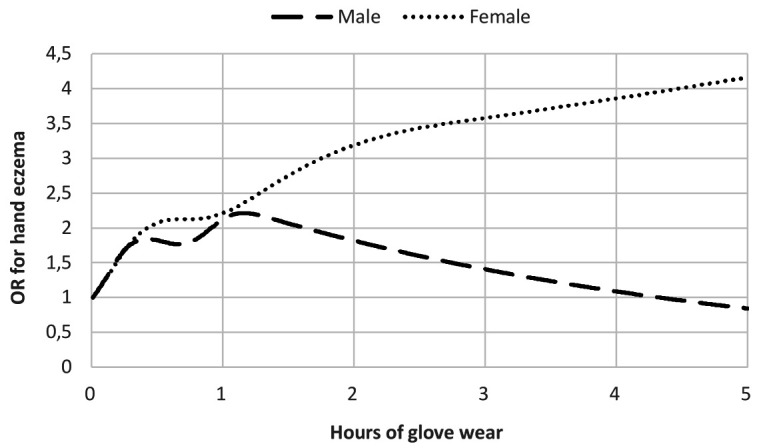
Risk (odds ratio) of hand eczema in men (N=3333) and women (N=11 908) according to a job exposure matrix derived estimate of average daily hours working with gloves

Figure 2 illustrates the dose–response relationship between amount of wet hands (average hours as a continuous measure) and risk of HE in females and males. OR for having HE doubled after 39 minutes for females and after 77 minutes for males. After two hours, the OR was 3.49 for females and 2.41 for males. Similarly, dose–response for the association between glove wear (average hours as a continuous measure) and risk (OR) of HE in females and males is illustrated in figure 3. Regarding exposure to glove wear, OR for having HE doubled after 27 minutes for females and after 55 minutes for males. After two hours OR was 3.19 for females and 1.82 for males.

## Discussion

Overall, we found OR of having HE significantly related to the extent of wet work, particularly among females. Dose–response curves for average time with wet hands and glove wear at work illustrated that OR for having HE doubled for both sexes earlier than the current definition of wet work ≥2 hours.

The risk of having HE in professions where 25–50% of workers are exposed to wet hands ≥2 hours/day was significantly increased and increased further in professions where 50–75% and 75–100% of workers are exposed to wet hands ≥2 hours/day. While the definition of wet work is widely accepted, and a clear association between wet work and HE is well established (7, 9, 10, 28–30), quantitative data on the dose–response relationship is sparse. Prior studies that have investigated the effect of water exposure to the skin, have shown that daily water exposure <1 hour/day does not irritate the skin (29, 31). Although based on small samples (N=21), *in vitro* pig skin and *in vivo* human skin, these findings may be used to support the present definition of wet work ≥2 hours/day in relation to HE, but no other studies have to our knowledge shown specific levels of cut-off. When adjusting for possible confounders such as age and atopic dermatitis, the risk of having HE for both females and males remained regarding wet hands (table 2a). However, when assessing the risk separately for males and females regarding glove wear (tables 2b and 2c), it becomes evident that the pattern differs for males, where we find that widespread use of gloves is not related to a significant increased risk of HE (Figure 3). The difference between the various confounder adjustments is that patch test and atopic dermatitis, do not seem to have much impact on risk of HE. These findings point towards that wet work is an independent risk factor of HE. Adjusting for socio-economic confounding attenuates risks somewhat, though when taking uncertainties in the analyses into account this does not change conclusions. Impact of confounding is similar in the sex stratified analyses.

In some male dominated professions, for example masonry and painting, a large number of workers wear non-occlusive gloves or a mix of occlusive and non-occlusive gloves. Despite the phrasing of the question regarding glove wear, including only occlusive glove material (plastic, rubber), a possible explanation for the difference between males and females may be that the question was understood to include gloves in general, thereby affecting responses from men more than women and resulting in a not-so-straightforward interpretation. Glove wear may also be for the protection against mechanical exposure, which was not investigated in this study. In this case, increased glove wear does not indicate increased wet work and the risk of HE (due to wet work) no longer applies.

Whether glove wear constitutes a risk exposure or a protective factor is still under discussion, and there are several studies with results pointing in both directions (32–37). Due to the present definition of wet work, where glove wear is included, we have chosen to maintain it as a such in our study.

Our findings illustrate that the risk of HE also increases when performing wet work <2 hours per day on average, which is the timeframe defining wet work today (figure 2) (11). This represents a current risk of overlooking both specific wet-work tasks as well as specific wet-work professions where this time definition of ≥2 hours/day is not obtained, but which however may still lead to HE. Our results were most significant for females, confirming previous studies describing the risk profile of wet-work professions (2, 10, 14, 38). A biological difference such as a higher susceptibility in female skin sensitivity has been excluded in several studies (39, 40), and the increased risk of HE found in females may therefore be solely related to exposure (41, 42).

### Strengths and limitations

To the best of our knowledge, this is the first study to present an exposure–response relationship between extent of wet work and the diagnosis HE. One of the strengths of our study is that it is based on independent sources of data, minimizing the risk of recall bias. We have been able to further strengthen this study with a recently developed wet-work-specific JEM, based on large nationwide representative survey data with a high participation rate (23). The group of patients comprising the case–referent population are appropriate for our aim due to the availability of both a HE diagnosis and patch test results, the latter to be used when checking for confounding. The risk of misclassification bias regarding validity of diagnoses is assumed to be low as a dermatologist diagnosed both the HE and facial eczema, and consequently also the absence of having HE. Although possible differential diagnosis does exist, this will only count for a few cases. Nevertheless, it is acknowledged that the validity of the diagnoses in the National Database of Contact Allergy has not been explicitly documented. The number of missing data on profession in this population was low, which further strengthens the study. We chose to use DISCO-codes from the year previous to the year of patch testing. The choice was based on the assumption that a high probability of workers either were in the same position the year of patch testing as the year before or a position in the same occupational category. This choice may have led to misclassification bias regarding workers who may have changed profession resulting in a likely attenuated estimate of the actual risk. The independent sources of data based on large and broad survey data strengthen the external validity of this study.

Apart from occupational exposure, the importance of wet work at home has been discussed in earlier studies (43, 44), and a positive relationship between occupational wet-work exposure and wet work at home has been found (22, 45) We accounted for this aspect by adjusting for resident children ≤4 years of age. However, information on other activities such as certain leisure activities eg, fishing and gardening were not accessible. Adjusting for confounding by socio-economic factors (residence, income level, educational level) tended to attenuate risk estimates, which might in part be explained by difference in health seeking behavior.

Other possible confounders of interest are some lifestyle factors. Tobacco smoking is a risk factor for HE with a strong gradient across professions and thus a likely important confounder (24). We accounted for confounding by smoking by use of a sex-, age- and calendar-time specific JEM which in large national samples predicts all-cause mortality and acute myocardial infarction independently of other risk factors (26, 46). In addition to tobacco smoking, which have been included as JEM-based estimates, other lifestyle factors such as exercise and level of stress could be of interest (47, 48).

Misclassification of exposure may arise when the average exposure at the group level is assigned to all individuals belonging to the group. This occurs when exposure data is based on a JEM, which per definition does not reflect any variation among individuals working in the same profession. The consequence of non-differential misclassification of exposure may be attenuated risk estimates which first of all is a problem in JEM based studies that contrary to our study are presenting null findings. However, to the extent that that the assigned average JEM-based values are valid, group-based exposure assessment is likely predominantly to be associated with a Berkson-type of error rather than a classical error which tends to have unbiased or less-biased associations but wider CI (49–51).

The potential risk of classifying exposure levels into broad groups is that we assume equal risk within these groups, which may underestimate the OR. However, our analysis of time of exposure is based on continuous exposure data and reaffirms the overall conclusions that increased exposure increases the risk of hand eczema.

In a recently published study comparing self-reported data to observational data on wet work (21), we found that professions with high wet-work prevalence overestimated duration of wet-work activities. This finding is in accordance with Jungbauer et al (15) and Anveden et al (17), who also found duration of wet work to be overestimated in self-reported studies. Future studies could consider that the increased risk of HE may occur at much shorter durations of wet work than our data show. This could support an even more restrictive approach towards possible legislation in the area of prevention of HE.

### Concluding remarks

Dose–response curves for wet work showed a significant risk of having HE even at an exposure level of ≤30 minutes on average of wet work/day. Our data confirmed a close association between wet work and HE and illustrated that exposure lasting less than the current definition of wet work (≥2 hours) may be of importance. Based upon the results of this study, this definition may need to be reevaluated.

Females had higher risk of HE compared to men when probability of having wet hands was <75%, but males had higher risk of HE compared to females when probability of wet hands was >75%

Regarding glove wear, females had higher risk of HE compared to men at all levels of glove wear when compared to the reference group.
